# Spatial Rearrangement and Mobility Heterogeneity of an Anionic Lipid Monolayer Induced by the Anchoring of Cationic Semiflexible Polymer Chains

**DOI:** 10.3390/polym8060235

**Published:** 2016-06-17

**Authors:** Xiaozheng Duan, Yang Zhang, Ran Zhang, Mingming Ding, Tongfei Shi, Lijia An, Qingrong Huang, Wen-Sheng Xu

**Affiliations:** 1 State Key Laboratory of Polymer Physics and Chemistry, Changchun Institute of Applied Chemistry, Chinese Academy of Sciences, Changchun 130022, China; xzduan@ciac.ac.cn (X.D.); rzhangciac@ciac.ac.cn (R.Z.); lljan@ciac.ac.cn (L.A.); 2 School of Business, Northeast Normal University, Changchun 130024, China; yzhang_nenu@hotmail.com; 3 Department of Food Science, Rutgers University, 65 Dudley Road, New Brunswick, NJ 08901, USA; qhuang@aesop.rutgers.edu; 4 James Franck Institute, The University of Chicago, Chicago, IL 60637, USA

**Keywords:** semiflexible polymers, anchoring, sequestration, mobility, anionic lipid, Monte Carlo

## Abstract

We use Monte Carlo simulations to investigate the interactions between cationic semiflexible polymer chains and a model fluid lipid monolayer composed of charge-neutral phosphatidyl-choline (PC), tetravalent anionic phosphatidylinositol 4,5-bisphosphate (PIP_2_), and univalent anionic phosphatidylserine (PS) lipids. In particular, we explore how chain rigidity and polymer concentration influence the spatial rearrangement and mobility heterogeneity of the monolayer under the conditions where the cationic polymers anchor on the monolayer. We find that the anchored cationic polymers only sequester the tetravalent PIP_2_ lipids at low polymer concentrations, where the interaction strength between the polymers and the monolayer exhibits a non-monotonic dependence on the degree of chain rigidity. Specifically, maximal anchoring occurs at low polymer concentrations, when the polymer chains have an intermediate degree of rigidity, for which the PIP_2_ clustering becomes most enhanced and the mobility of the polymer/PIP_2_ complexes becomes most reduced. On the other hand, at sufficiently high polymer concentrations, the anchoring strength decreases monotonically as the chains stiffen—a result that arises from the pronounced competitions among polymer chains. In this case, the flexible polymers can confine all PIP_2_ lipids and further sequester the univalent PS lipids, whereas the stiffer polymers tend to partially dissociate from the monolayer and only sequester smaller PIP_2_ clusters with greater mobilities. We further illustrate that the mobility gradient of the single PIP_2_ lipids in the sequestered clusters is sensitively modulated by the cooperative effects between anchored segments of the polymers with different rigidities. Our work thus demonstrates that the rigidity and concentration of anchored polymers are both important parameters for tuning the regulation of anionic lipids.

## 1. Introduction

A deep understanding of the interactions between peripheral cationic bio-macromolecules and anionic lipid membranes is crucial for many cellular activities [1,2], including ion-channel activation, vesicle trafficking, cellular apoptosis, enzyme activation, *etc.* The key component in the lipid membrane that launches the anchoring of bio-macromolecules is the phosphatidylinositol 4,5-bisphosphate (PIP_2_) lipid, which has a valence of −4 at pH = 7.0 [3], and therefore enables the electrostatic anchoring of proteins or polypeptides with a cluster of polybasic residues. Despite the fact that PIP_2_ lipids constitute only about 1% of all phospholipids in the plasma membrane, this versatile lipid species is essential for nearly all signaling functions in living cells [1,4,5]. As another important functional phospholipid, phosphatidylserine (PS) has a single net negative charge and constitutes about 10%–20% of all lipids. The univalent PS lipids can thus regulate the surface charge density of the membrane to induce the anchoring of cationic polymers, thereby controlling some specific physiological activities [2,6].

The complex coacervates of charged macromolecules and liposomes have been extensively studied via a variety of experimental techniques [1,4,6,7,8,9,10], such as X-ray scattering, transmission electron microscopy, fluorescence resonance energy transfer measurements, *etc.* These studies have demonstrated that the anchored charged macromolecules can adjust the fraction of free anionic lipids and cause the mobility heterogeneity of the membrane through the electrostatic sequestration of the anionic lipids, thereby offering useful information for the development of biological and medical applications [11,12,13,14,15,16]. However, it remains a significant challenge to investigate the macromolecule/membrane complexes at a molecular scale via experimental approaches, due to the hydrolysis and fast turnover of the anionic lipids *in vivo* [1]. In particular, it is generally difficult to investigate the influence of each individual molecular parameter on the interactions between charged macromolecules and liposomes experimentally. Hence, computational and theoretical methods provide complementary tools for better understanding the mechanisms of these interfacial interactions. For example, a recent study based on the atomic model [17] indicates that the anchored polypeptides can cause the sequestration and dynamic restriction of anionic lipids, which theoretically rationalizes the experimental measurements via microscopy. The coarse-grained models have also been widely employed in simulations to elucidate the biophysical properties of the macromolecule/membrane complex coacervates [18,19,20,21,22,23,24,25,26,27,28]. Results from these models have demonstrated that several important molecular factors—such as the shape, size, and ionization degree of the peripheral macromolecules, and the charge of the lipids—all affect the stability, structure, and dynamics of the complexes significantly. While the intrinsic rigidity evidently represents an important characteristic feature of the peripheral cationic macromolecules, the influence of this important molecular parameter on the structure and dynamics of membrane/macromolecule complexes has been addressed in a limited way in previous simulations [1,22]. Moreover, the concentration of peripheral macromolecules often varies considerably from one region to another *in vivo*; e.g., the growth-associated protein 43 exhibits a higher concentration in the axonal growth cones of neurons and some other regions [1,29]. Therefore, it is then natural to ask how the rigidity and concentration of polymers determine their anchoring on lipid membranes, and how these factors affect the resulting properties of macromolecule/membrane complexes.

Our recent work based on Monte Carlo (MC) simulations has investigated the anchoring of a single flexible cationic polymer onto a fluid monolayer composed of charge-neutral and anionic lipids [30,31,32]. Our simulations show that the cationic polymer can cause significant sequestration of the anionic lipids and reduce mobility of the lipids. Our work also indicates that the sequestration and mobility of the anionic lipids are strongly dependent on the charge of lipid headgroups and the ionic strength of the solution. These trends are in qualitative agreement with recent experimental studies [7]. Our previous work also addresses the effects of chain rigidity on the stability and corresponding entropy–energy balance of the polymer/monolayer complex [33,34], where only a single polymer chain anchored on the lipid membrane is considered. In this paper, we further extend our study to the anchoring of multiple cationic polymers with variable chain stiffness onto a fluid mixed lipid monolayer, and therefore, we explicitly explore how chain rigidity and polymer concentration affect the properties of macromolecule/membrane complexes with a particular focus on the sequestration and mobility heterogeneity of the anionic lipids.

The rest of the paper is organized as follows. In Section 2, we introduce the MC model and provide a brief introduction to the simulation details. In Section 3, we discuss the interactions between the anchored polymers and the monolayer and analyze the effects of polymer rigidity and concentration on the structure and mobility of the polymers/monolayer complex. In Section 4, we draw some concluding remarks.

## 2. Model and Simulation Method

### 2.1. Monte Carlo Model

Our Monte Carlo (MC) model of the polymers/monolayer complex has been described in detail in our previous work [30,31,32,33,34], so we briefly introduce this model here. We employ the freely-jointed chain model to coarse-grain the cationic polymers [35]. Each polymer chain includes *N* = 20 connected segments, with each segment taking a positive charge of *Z*_s_ = +1 at its center. All segments have the same diameter of *d* = 8.66 Å. The lipid monolayer is composed of 50 × 50 lipid headgroups, which are modeled as 2500 hexagonal closely-packed disks (also with a diameter of *d* = 8.66 Å) on an impenetrable plane at *z* = 0 of the simulation box. The monolayer contains the charge-neutral phosphatidyl-choline (PC), the univalent PS, and the tetravalent PIP_2_ lipid molecules. Driven by the biophysical interests [1,22], we consider the following compositions of the monolayer in this work: PC:PS:PIP_2_ = 98:1:1, PC:PIP_2_ = 99:1 and PC:PS:PIP_2_ = 89:10:1. While the composition with PC:PIP_2_ = 99:1 permits investigations of the interactions between anchored polymers and the anionic tetravalent PIP_2_ lipids, we can further compare the sequestration and mobility heterogeneity of both PS and PIP_2_ lipids caused by polymer anchoring for PC:PS:PIP_2_ = 98:1:1. The case with PC:PS:PIP_2_ = 89:10:1 represents the membrane with more realistic lipid compositions, as discussed in Refs. [22,24]. However, since the general trends of the examined properties are quite similar for different compositions, we mainly focus on the case of PC:PS:PIP_2_ = 98:1:1 and briefly discuss the results for the other compositions.

We express the potential energy of the system in the form of

(1) $$U=U_{\mathrm{NB}}+U_{\mathrm{bend}}$$

where *U*_NB_ and *U*_bend_ designate the contributions arising from non-bonded and angular interactions, respectively. Since we fix the bond length of the polymers as a constant of *d*, the contribution from bonded interactions to the potential energy is simply zero. The non-bonded contribution *U*_NB_ contains the hard sphere potential (*U*_HS_) between polymer segments and the electrostatic potential (*U*_E_) between charged species,

(2) $$U_{\mathrm{NB}}=U_{\mathrm{HS}}+U_{E}$$

We consider the polymers/monolayer complex in a symmetric univalent salt aqueous solution, and further account for the electrostatic interactions between charged species through Debye–Hückel theory,

(3) $$U_{E}=\frac{1}{2}k_{B}T\sum_{m\neq n}Z_{m}Z_{n}l_{B}\frac{\exp(-l_{D}r_{\mathrm{mn}})}{r_{\mathrm{mn}}}$$

where *Z*_m_ and *Z*_n_ are the valences of the charges *m* and *n*, *r*_mn_ is the distance between charges *m* and *n*, *l*_B_ is the Bjerrum length taken as 7.14 Å in aqueous solution at 278 K, and *l*_D_ is the inverse Debye screening length. We adopt the Debye screening length (*l*_D_^−^) to account for the screen effects from the solution,

(4) $${l_{D}}^{-}=\frac{1}{\sqrt{1000e^{2}N_{A}\sum_{i}\frac{Z_{i}^{2}C_{i}}{\varepsilon_{0}\varepsilon_{r}k_{B}T}}}$$

where *e* is the elementary charge, *N*_A_ is Avogadro’s constant, *Z*_i_ is the valence of the salt ions, *C*_i_ is the salt concentration of the solution, and ε_r_ is the dielectric permittivity, which equals ~78.5 in the salt solution and ~2 within the monolayer [23,24]. The energy and distance are expressed in units of *k*_B_*T* and *d*, respectively.

We further adopt a harmonic bending potential *U*_bend_ to adjust the intrinsic rigidity of the cationic polymers,

(5) $$U_{\text{bend}}=\kappa k_{B}T\sum_{i=2}^{N-1}{\left(\alpha_{i}-\alpha_{0}\right)}^{2}$$

where α_i_ denotes the angle specified by the connected segments *i* + 1, *i*, and *i* − 1, α_0_ equals π, and the chain rigidity parameter *κ* tailors the strength of angular interactions and hence the degree of chain rigidity. It is then expected that elevating *κ* leads to an increase in the persistence length (*L*_p_), which we analyze below by computing this quantity from the bond angle correlation function in our simulations. While our model is clearly rather idealized, we expect that it can capture some qualitative trends that will be useful for better understanding the interactions between bio-macromolecules and lipid membranes.

### 2.2. Simulation Details

We perform simulations in an NVT ensemble. Periodical boundary conditions are applied in the *x* and *y* directions, and the *z* direction is taken as infinite. We implement the relaxation of the polymers via kink-jump, crankshaft, translation and pivot movements [36], and employ the Kawasaki algorithm [25,37,38] to model the lipid motions. Each MC step includes the trial movements of all polymer segments and charged lipids in accordance with the Metropolis algorithm [39]. In each run, we put the cationic polymers onto the monolayer and generate the anionic lipids on the monolayer stochastically. We first perform 10^6^ MC steps for athermal relaxation in order to eliminate the artificial effects from the initial configuration, and the polymers are controlled to relax above the monolayer at this stage. We then perform 2 × 10^6^ MC steps for equilibration, and another 2 × 10^6^ MC steps for data analysis. For each state point, we run 20 parallel simulations.

We vary the rigidity parameter *κ* of the polymers from 0 to 100, a range of values that allows for the simulation of fully flexible chains (*κ* = 0), chains with intermediate rigidity (0 < *κ* ≤ 20), and chains with high rigidity (*κ* > 20). The effect of polymer concentration is explored by adjusting the number *N*_c_ of polymer chains from 2, 4, 8, to 16. These chain numbers can be converted into polymer concentrations (*C*_p_ = *N*_c_/*A*_m_) as 0.00092, 0.00185, 0.0037, to 0.0074 [*d*^−2^], where the concentration is measured via the number of chains per unit area. Since our interest here is to study the polymers/monolayer interactions, we mainly consider the cases with the salt concentration of *C_i_* = 0.01 M, for which the polymers anchor onto the monolayer. We only briefly discuss the salt effect of the solution on the stability and mobility of the complex.

To characterize the stiffness of the polymer chains, we calculate the bond angle correlation function BAC(*D*_C_)~e^(−*D*C/*L*p)^ [40] to extract the persistence length *L*_p_ of the cationic polymers, where *D*_C_ denotes the contour distance along the polymer chain and *L*_p_ includes the electrostatic contribution (*L*_e_) and the intrinsic part (*L*_0_). In Table 1, we summarize the persistence length *L*_p_ of the polymers for various *κ* at different salt concentrations *C_i_*. We see that *L*_p_ increases with *κ* for fixed *C*_i_, as expected. Moreover, the salt concentration *C*_i_ exerts a strong impact on *L*_p_.

## 3. Results and Discussion

### 3.1. Sequestration of Anionic Lipids Underneath Anchored Polymers

**Figure 1:**
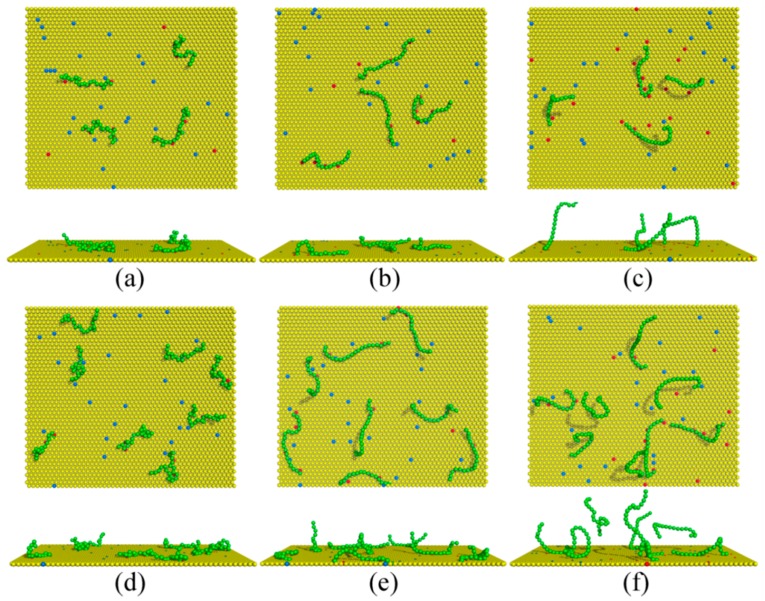
Equilibrated simulation snapshots of the polymers/monolayer complexes for the monolayer composition with PC:PS:PIP_2_ = 98:1:1 for (**a**–**c**) *C*_p_ = 0.00185 and (**d**–**f**) *C*_p_ = 0.0037. Green spheres denote the polymer segments; yellow, blue, and red spheres represent the PC, PS, and PIP_2_ lipid headgroups. The chain rigidity parameter *κ* of the polymers is set as (**a**,**d**) 0, (**b**,**e**) 10, and (**c**,**f**) 100, respectively.

As an illustration of the membrane heterogeneity caused by the polymer anchoring, Figure 1 presents representative simulation snapshots of the complexes after equilibration for various chain rigidity parameters *κ* at two polymer concentrations. This figure clearly shows that both polymer rigidity and concentration influence the properties of the polymers/membrane complexes, which we analyze below in detail.

**Figure 2:**
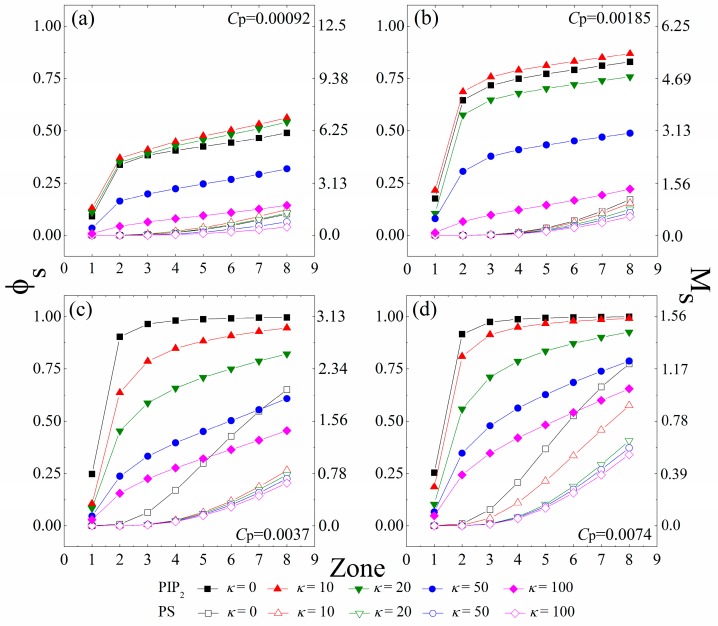
Fraction (φ_s_) of the anionic lipids (PIP_2_, solid symbols; PS, vacant symbols) sequestered in the interaction zones of all anchored polymers, and the number (*M*_s_) of the anionic lipids (PIP_2_, solid symbols; PS, vacant symbols) sequestered in the zones underneath each anchored polymer. The polymer concentration is set as (**a**) *C*_p_ = 0.00092; (**b**) *C*_p_ = 0.00185; (**c**) *C*_p_ = 0.0037 to (**d**) *C*_p_ = 0.0074. The chain rigidity parameter (*κ*) of the polymers varies from 0 to 100. The results shown are for PC:PS:PIP_2_ = 98:1:1. PIP_2_: phosphatidylinositol 4,5-bisphosphate; PS: Phosphatidylserine; PC: Phosphatidyl-choline.

Since each charged polymer/lipids complex minimizes its energy at the isoelectricity [1], a certain amount of anionic lipids tend to sequester around the anchored cationic polymers to achieve the charge matching. To differentiate the lipids “sequestered” by the anchored polymers from those freely diffusing on the monolayer, we use the same method as in our previous work [31,32,34], which has shown to be an efficient procedure to characterize the properties of the oppositely-charged species bounded by the charged polymers [41]. Here, we briefly introduce this method. We first define eight interaction zones on the monolayer underneath each anchored polymer. The *k*^th^ zone—with its size determined by the conformation of the anchored polymers in each MC step—denotes the area in a distance less than *k* × *d* (8.66 Å) to each segment center of the anchored polymers. We then examine the system in every 50 MC steps and consider an anionic lipid “sequestered” in the *k*^th^ zone if it remains within the zone for this time interval. We further define the lipids sequestered in each zone as a lipid micro-domain, and calculate the number of the sequestered anionic lipids in these micro-domains. In Figure 2, we present the fraction (φ_s_) of the sequestered lipids in the interaction zones of all anchored polymers, and the corresponding number (*M*_s_) of the sequestered lipids in the zones underneath each anchored polymer for various polymer concentrations and chain rigidity parameters.

Figure 2a,b reveal an apparent concentration gradient of the tetravalent PIP_2_ lipids caused by polymer anchoring at low polymer concentrations (*C*_p_ = 0.00092 and 0.00185). For the cases with *κ* = 0 (fully flexible chains), most of the sequestered PIP_2_ lipids stay in the 2nd interaction zone of the anchored polymers, whereas from the 2nd to the 8th zone, the amount of the segregated PIP_2_ lipids grows slowly. Due to the lower demixing entropy loss of PIP_2_ lipids for sequestration, these tetravalent lipids preferably sequester around the anchored polymers, which competitively inhibit the binding of the PS lipids. Therefore, we do not observe the PS sequestration from the 1st to the 4th zone, and only a few PS lipids randomly appear in the 5th to the 8th zones. By increasing *κ* from 0 (fully flexible chains, black squares) to 10 (chains with intermediate rigidity, red triangles), we find a slight growth in φ_s_, indicating an increased amount of the PIP_2_ lipids sequestered underneath the anchored polymers. However, by further increasing *κ* to 100 (chains with high rigidity, purple diamonds), the amount of the PIP_2_ lipids sequestered in each interaction zone significantly decreases. Each polymer anchors on the monolayer when the electrostatic energy gain from neutralization exceeds the increase in the entropy loss of the system. The enhancement of chain rigidity forces the anchored polymers to flatten onto the monolayer, which contributes to the polymer anchoring through reducing its conformational entropy loss. Meanwhile, the demixing entropy loss of the anionic lipids sequestered underneath the anchored stiffer polymers is significantly enlarged, which in turn promotes dissociation of the polymers from the monolayer. This competition thus appears to rationalize our observation that the strength of interactions between the polymers and the monolayer exhibits a non-monotonic dependence on the intrinsic rigidity of polymer chains. Hence, our work reveals that the PIP_2_ lipids exhibit maximum sequestration for the anchoring of chains with intermediate rigidity (*κ* = 10 and 20) at low polymer concentrations.

Figure 2c,d indicate that the fraction φ_s_ of the total sequestered PIP_2_ lipids significantly increases with increasing *C*_p_, but each chain sequesters a lower amount of the PIP_2_ lipids (indicated by a decrease in *M*_s_) because of the inter-polymer competitions at relatively high polymer concentrations. Under these conditions, the fully flexible polymer chains can slave nearly all PIP_2_ lipids in the 3rd zones (*i.e.*, φ_s_ ≈ 1.0) and a number of PS lipids also sequester in the 4th to 8th zones, indicating the aggregation of these univalent lipids around the polymer/PIP_2_ complexes. More intriguingly, the decreased electrostatic energy gains of each polymer/PIP_2_ complex cannot conquer the entropy loss of the system, because of the enhanced inter-polymer competitions for the anchoring of chains with intermediate rigidity (*κ* = 10 or 20). Therefore, we do not observe the non-monotonic dependence of the strength of interactions between the polymers and the monolayer on the polymer rigidity at relatively high polymer concentrations (Figure 2c,d). In these cases, the amount of the sequestered PIP_2_ lipids significantly decreases with increasing *κ*.

**Figure 3:**
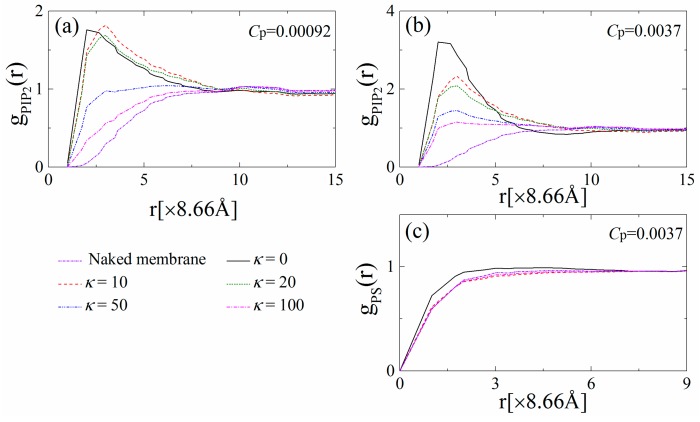
Radial distribution function of the PIP_2_ lipids for (**a**) *C*_p_ = 0.00092 and (**b**) *C*_p_ = 0.0037 for various chain rigidity parameters *κ*; (**c**) Radial distribution function of the PS lipids for *C*_p_ = 0.0037. The results shown are for PC:PS:PIP_2_ = 98:1:1.

Figure 3 displays the radial distribution functions (RDF) for PIP_2_–PIP_2_ and PS–PS lipids, which are denoted as *g*_PIP2_(*r*) and *g*_PS_(*r*), respectively. Figure 3a,c indicate that both *g*_PIP2_(*r*) and *g*_PS_(*r*) increase with *r* and gradually reach unity for the naked monolayer when polymer anchoring does not occur (purple dashed-dotted lines). When the polymer concentration is low (e.g., *C*_p_ = 0.00092 in Figure 3a), the flexible polymers anchor onto the monolayer and cause substantial sequestration of the PIP_2_ lipids, resulting in the formation of an apparent peak in *g*_PIP2_(*r*) at *r* = 2 (×8.66 Å). With *κ* increasing to 10 (chains with intermediate rigidity), the peak position shifts from *r* = 2 to *r* = 3 (×8.66 Å), and the *g*_PIP2_(*r*) exhibits an increase at 3 < *r* < 8 (×8.66 Å), indicating that the anchored polymers exhibit a more stretched structure and sequester a few more PIP_2_ lipids in a less-compact way than the flexible ones. For chains with high rigidity (e.g., *κ* = 50 or 100), the polymers tend to dissociate from the monolayer, and thus fewer PIP_2_ lipids are sequestered, leading to a drastic decrease in *g*_PIP2_(*r*) at *r* < 10 (×8.66 Å). We also find that the corresponding *g*_PS_(*r*) at *C*_p_ = 0.00092 exhibits nearly the same behavior as the naked monolayer (data not shown), which illustrates that the univalent PS lipids do not sequester around the polymers in the presence of tetravalent PIP_2_ lipids.

By increasing the polymer concentration to *C*_p_ = 0.0037, Figure 3b shows that the peak values of *g*_PIP2_(*r*) significantly increase, indicating the increase in the total amount of the PIP_2_ lipids sequestered by the concentrated anchored polymers. For the anchoring of flexible polymers, when the 1% PIP_2_ lipids are not sufficient to neutralize the cationic polymers, the PS lipids accumulate around polymer/PIP_2_ complexes, and therefore *g*_PS_(*r*) at *r* < 4 (×8.66 Å) slightly increases (the solid line in Figure 3c). Further, increasing polymer chain rigidity results in the partial dissociation of the polymers and the decay of the PIP_2_ sequestration, and therefore, *g*_PIP2_(*r*) at small *r* significantly decreases. In addition, even if the total charge amount of the polymers exceeds those of the PIP_2_ lipids, we do not observe the clustering of the PS lipids, and *g*_PS_(*r*) exhibits similar behavior as that for the naked monolayer (e.g., see the red dashed line in Figure 3c).

For comparison, we also analyze the variations of φ_s_ and *M*_s_ with *κ* and *C*_p_ for the system with PC:PIP_2_ = 99:1 as well as g_PIP2_(*r*) and *g*_PS_(*r*) for the systems with PC:PIP_2_ = 99:1 and PC:PS:PIP_2_ = 89:10:1 (data not shown). For PC:PIP_2_ = 99:1, the PIP_2_ sequestration exhibits a similar dependence on the rigidity and concentration of the anchored polymers as the system with PC:PS:PIP_2_ = 98:1:1. For PC:PS:PIP_2_ = 89:10:1, we also find the similar dependences of PIP_2_ and PS redistribution on the rigidity and concentration of the anchored polymers as those for PC:PS:PIP_2_ = 98:1:1. In addition, the sizable pools of the univalent PS lipids promote the polymer anchoring, which in turn enhances the sequestration of the anionic lipids, and therefore, we observe larger values of *g*_PIP2_(*r*) for the stiffer chains compared to those for PC:PIP_2_ = 99:1 and PC:PS:PIP_2_ = 98:1:1.

### 3.2. Distribution of the Anchored Polymer Segments

**Figure 4:**
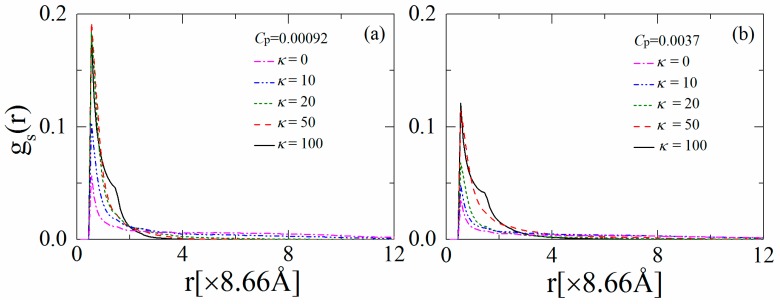
Distribution function for segments of the anchored polymers on the monolayer for various polymer chain rigidity parameters *κ* for (**a**) *C*_p_ = 0.00092 and (**b**) *C*_p_ = 0.0037. The results shown are for PC:PS:PIP_2_ = 98:1:1.

We now focus on the properties of the anchored polymers in order to further elucidate the interactions between the polymers and the anionic lipids. In particular, we examine the distribution function *g*_s_(*r*) for the segments of the anchored polymers above the monolayer surface. Figure 4a indicates a shoulder peak in *g*_p_(*r*) for the fully flexible chains when the polymer concentration is low (*i.e.*, *C*_p_ = 0.00092), which is due to the presence of segments in the loops and tails of the anchored polymers. The observation of a shoulder peak in *g*_p_(*r*) corresponds well with the previous simulation results for the adsorption of charged polymers on oppositely-charged interfaces [42]. Moreover, the chains with intermediate rigidity (*κ* = 10) flatten onto the monolayer, resulting in a slight increase in the contact value of *g*_p_(*r*) at *r* < 1 (×8.66 Å), but the decay of the shoulder peak at 1 < *r* < 2 (×8.66 Å). This result implies that the increased cooperative effects between the anchored cationic segments can enhance the sequestration and restriction of the anionic lipids. By further increasing *κ* from 20 to 100, the anchored polymers tend to dissociate from the monolayer, which leads to an apparent decrease in the peak values of *g*_p_(*r*) and an increase in *g*_p_(*r*) at *r* > 2 (×8.66 Å). In Figure 4b, we show the results of *g*_p_(*r*) for *C*_p_ = 0.0037. The polymers contend for anchoring onto the monolayer at this concentration, and therefore, we observe an apparent decrease in the contact value of *g*_p_(*r*) compared to the results in Figure 4a. The enhancement of the polymer chain rigidity results in the partial dissociation of the anchored polymers, and hence, the contact value of *g*_p_(*r*) decreases with increasing *κ—*indicating that the polymers exhibit the brush-like conformations. This analysis supports our earlier observation that the partially dissociated stiffer polymers sequester smaller lipid clusters at relatively high polymer concentrations.

### 3.3. Restricted Mobility of the Polymer/Lipids Complexes

Prior experimental studies have demonstrated that the anchored cationic polymers can significantly slow down the mobility of the sequestered lipids, which in turn exerts a restriction on the dynamics of the anchored polymers [9,23]. To qualitatively capture the mobility of the polymers/monolayer complexes, we calculate the mean-square-displacement (MSD) of each lipid micro-domain as well as the single sequestered lipids in it in every 50 MC steps, denoted as *m*_d_ and *m*_l_, respectively. We also analyze the corresponding MSD of the center of mass of the anchored polymers, denoted as *m*_pc_. Our previous simulation results [31,32] show that the cationic polymer can cause significant sequestration of the anionic lipids and thus reduce their translational mobility, which is in qualitative agreement with recent experimental measurements [7]. Moreover, we illustrate that the mobility of a flexible cationic polymer anchored on the monolayer satisfies the scaling of *m*_p_~*N*^−1^ (where *N* is the polymerization degree of polymers). This scaling was also observed in a previous experimental study [43]. This implies that our simulations may provide a reliable tool for understanding the mobility of polymers/lipids complexes in the equilibrium state, at least in a qualitative manner despite the rather idealized nature of our model. We now discuss the effects of the rigidity and concentration of the anchored polymers on the restricted mobility of the polymers/monolayer complexes.

**Figure 5:**
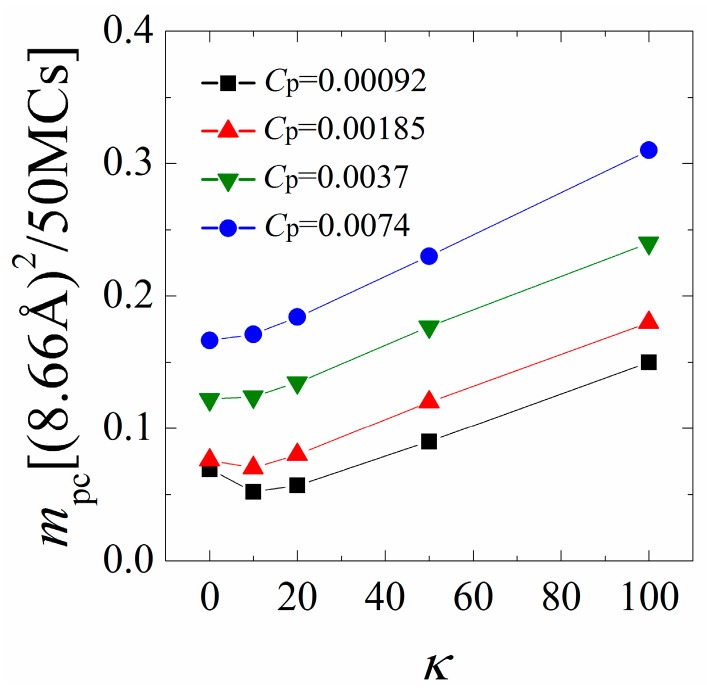
Mean-square-displacement (MSD) of the center of mass of the anchored polymers calculated in every 50 Monte Carlo (MC) steps as a function of chain rigidity parameter *κ* for various polymer concentrations. The results shown are for PC:PS:PIP_2_ = 98:1:1.

Figure 5 shows *m*_pc_ as a function of the chain rigidity parameter *κ* for various polymer concentrations *C*_p_. Our earlier analysis indicates that the polymers/monolayer interactions are strengthened with increasing *κ* from 0 to 10 when the polymer concentration is low. As a consequence, we find that *m*_pc_ first slightly decreases with increasing chain rigidity for low polymer concentrations, indicating a slower mobility of the anchored polymers. By further increasing *κ* from 20 to 100, the cationic polymers tend to dissociate from the monolayer and protrude longer tails into the solution, and thus exhibit a faster mobility, as evidenced by a significant increase in *m*_pc_. The variation of *m*_pc_ with *κ* changes both quantitatively and qualitatively when the polymer concentration is high. In this case, the inter-chain competitions promote the dissociation of each polymer from the monolayer; therefore, we observe a significant increase in *m*_pc_ as compared to the results at low polymer concentrations. In addition, the interactions between the monolayer and the polymers are significantly weakened with increasing the chain rigidity at high polymer concentrations, where the mobility of the anchored polymers monotonically increases with increasing the chain rigidity.

**Figure 6:**
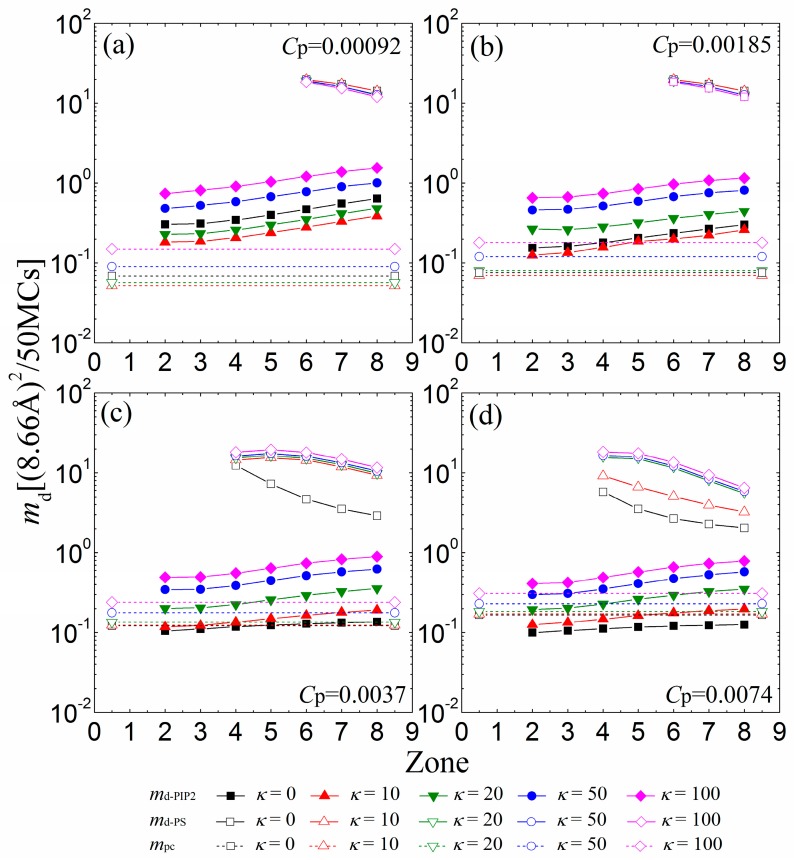
MSD of the center of mass of the sequestered PIP_2_ micro-domains (*m*_d-PIP2_, solid symbols and solid lines) and PS micro-domains (*m*_d-PS_, vacant symbols and solid lines) calculated in every 50 MC steps for various chain rigidity parameters *κ* for (**a**) *C*_p_ = 0.00092; (**b**) *C*_p_ = 0.00185; (**c**) *C*_p_ = 0.0037; and (**d**) *C*_p_ = 0.0074. Vacant symbols with dashed lines indicate *m*_pc_ of the anchored polymers. The results shown are for PC:PS:PIP_2_ = 98:1:1.

We now investigate the mobility of the sequestered anionic lipid clusters underneath the anchored cationic polymers. Figure 6a indicates that the mobility of lipid clusters enhances from the 2nd to the 8th zone at low polymer concentrations (*C*_p_ = 0.00092), a trend that arises because the electrostatic attractions from the anchored cationic polymers weaken from the 2nd to the 8th zone at such concentrations. The PIP_2_ lipids sequestered by anchored polymers with *κ* = 10 become more restricted than by flexible polymers (*κ* = 0), as evidenced by a corresponding decrease in *m*_d-PIP2_. By increasing *κ* from 20 to 100, we observe that the PIP_2_ clusters also exhibit a faster mobility. This result accords well with previous MC simulation work [23], which has shown that the electrostatically-bound anionic PIP_2_ clusters migrate with the cationic polymers together. Recent experiments have shown the trapping of PIP_2_ lipids by peripheral cationic polymers and emphasized the mobility coupling between polymers and PIP_2_ lipids [7,9]. Our simulations are also in good agreement with these experimental results. Figure 6b indicates that increasing the polymer concentration to *C*_p_ = 0.00185 leads to a decrease in the mobility of the sequestered PIP_2_ lipid cluster. The increase in *C*_p_ enhances the competition between the anchored polymers, resulting in the formation of the smaller sequestered PIP_2_ lipid clusters. These smaller clusters can only move in the smaller interaction zones underneath the partially adsorbed polymers, and their mobility is more confined—as evidenced by a decrease in *m*_d-PIP2_. Due to the enhanced competitions between anchored polymers, the chains with *κ* = 20 exert weaker attractions on the monolayer than the flexible ones for *C*_p_ = 0.00185, which results in the formation of smaller PIP_2_ clusters with a decreased mobility.

For *C*_p_ = 0.0037 (Figure 6c) and *C*_p_ = 0.0074 (Figure 6d), the competitions among the anchored polymers are significantly strengthened, which leads to the further shrinkage of the sequestered PIP_2_ lipid clusters underneath fewer anchored segments of each polymer. These small lipid clusters are firmly restricted by the reduced anchored segments of the polymers, and therefore, we observe a decrease in the mobility of each PIP_2_ cluster compared with the cases for lower polymer concentrations. Importantly, the polymers with intermediate rigidity no longer exert stronger adsorption on the monolayer than the flexible ones because of the inter-polymer competitions; hence, the mobility of the PIP_2_ clusters exhibits a monotonic dependence on the polymer chain rigidity. For sufficiently high polymer concentrations, only the polymers with small rigidity parameters (e.g., *C*_p_ = 0.0037 and *κ* = 0 in Figure 6c; *C*_p_ = 0.0074 and *κ* ≤ 10 in Figure 6d) can overcharge the PIP_2_ lipids and further restrict the PS lipids (see the vacant squares and triangles with solid lines in Figure 6c,d), whereas the stiffer polymer chains cannot firmly adsorb on the monolayer and barely affect the mobility of the PS lipids. Our results imply that the polymers with high rigidity can serve to sequester and differentiate the multivalent lipids from the univalent ones through the electrostatic anchoring at sufficiently high polymer concentrations.

### 3.4. Mobility Gradient in Lipid Clusters

**Figure 7:**
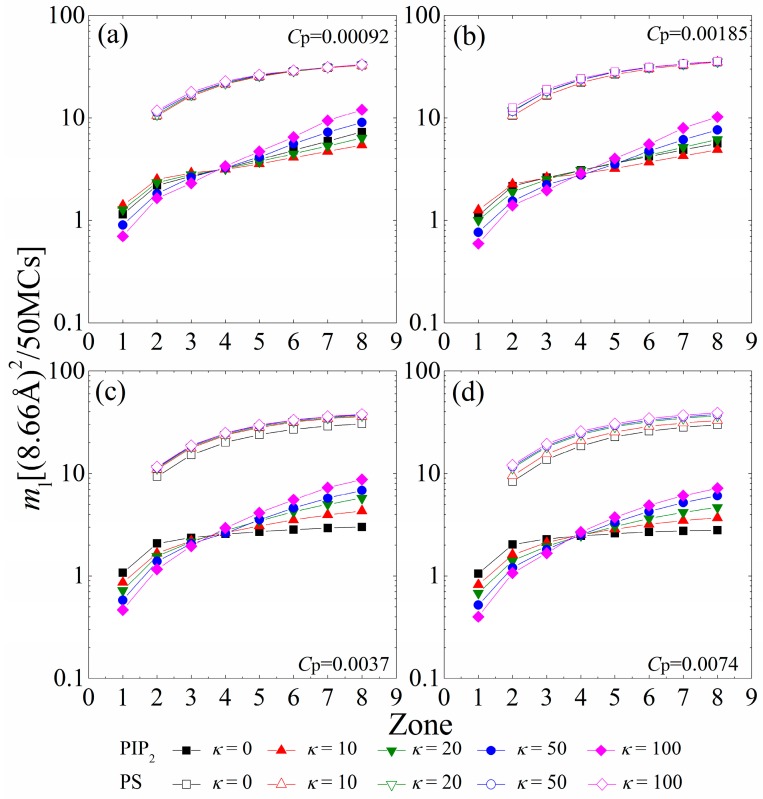
MSD of single PIP_2_ (*m*_l-PIP2_, solid symbols and solid lines) and single PS (*m*_l-PS_, vacant symbols and solid lines) sequestered in each interaction zone calculated in every 50 MC steps for various chain rigidity parameters *κ* for (**a**) *C*_p_ = 0.00092; (**b**) *C*_p_ = 0.00185; (**c**) *C*_p_ = 0.0037; and (**d**) *C*_p_ = 0.0074. The results shown are for PC:PS:PIP_2_ = 98:1:1.

To analyze the mobility gradient of the anionic lipids within each sequestered lipid cluster, Figure 7 displays the MSD of single PIP_2_ (*m*_l-PIP2_) and PS (*m*_l-PS_) lipids confined in the interaction zones underneath the anchored polymers. For the polymer concentration of *C*_p_ = 0.00092 shown in Figure 7a, the connected anchored segments of each polymer form several interaction zones, where the sequestered lipids can freely hop on different sites with the similar electrostatic fields. At this polymer concentration, chains with intermediate rigidity (*κ* = 10 or 20) tend to flatten onto the monolayer. The increased anchored segments of these chains create larger electrostatic homogeneous interaction zones on the monolayer. Therefore, the single PIP_2_ lipids restricted in the larger 1st or 2nd zones of these chains can freely hop on any site in these larger 1st or 2nd zones, and exhibit a faster mobility (e.g., filled red triangles in Figure 7a) than the ones confined within the smaller 1st or 2nd zones created by the lessened anchored segments of the flexible polymers (filled black squares in Figure 7a). In addition, the stronger cooperative effects between the increased cationic segments are enhanced in the larger zones (e.g., the 6th to the 8th) of the polymers with these intermediate rigidities. The PIP_2_ lipids confined in these larger zones (e.g., the 6th to the 8th) encounter stronger restrictions from the increased anchored cationic segments of the flattened polymers with intermediate rigidity, and exhibit a slower mobility (e.g., filled red triangles in Figure 7a) than those confined in the corresponding zones of flexible polymers (e.g., filled black squares in Figure 7a). Due to the decay in the cooperative effects between the lessened anchored segments, the PIP_2_ lipids sequestered in the 1st or 2nd zones suffer stronger restrictions and exhibit a slower mobility for the polymers with high rigidity (*κ* ≥ 50), whereas those sequestered in larger zones display a faster mobility, indicating a sharper mobility gradient of the PIP_2_ clusters. By increasing the polymer concentration to *C*_p_ = 0.00185 (Figure 7b), we observe that the mobility of single PIP_2_ lipids exhibits a similar dependence as the case for *C*_p_ = 0.00092 (Figure 7a). The enhanced competitions between anchored polymers (*C*_p_ = 0.00185) causes the reduced anchored segments for *κ* = 20 to exhibit weaker cooperative effects than those for *κ* = 0. Therefore, the PIP_2_ lipids sequestered in the 1st or 2nd zones exhibit a slower mobility, while those sequestered in larger zones display a faster mobility.

For higher polymer concentrations (e.g., *C*_p_ = 0.0037 and *C*_p_ = 0.0074, shown in Figure 7c,d), the polymers sequester nearly all PIP_2_ lipids. Meanwhile, the anchored polymers tend to exhibit a brush-like structure because of the enhanced inter-chain competitions, which greatly diminishes the cooperative effects between anchored segments. In each polymer/PIP_2_ complex, the fewer anchored segments exert stronger restrictions on the lessened PIP_2_ lipids; therefore, the mobility of the PIP_2_ lipids confined in each zone presents a decrease compared to the case for low polymer concentrations. The cooperative effects between the lessened anchored segments weaken with increasing chain rigidity, which results in the sharper mobility gradient of the sequestered PIP_2_ lipids. In addition, we only find a significant decrease in *m*_l-PS_ for *κ* = 0 at *C*_p_ = 0.0037 and *κ* ≤ 10 at *C*_p_ = 0.0074. This also demonstrates that the PS lipids can only sequester around the anchored flexible polymers, even if the total charge amount of the polymers is larger than that of the PIP_2_ lipids.

Our results thus illustrate the complicated mobility behavior of the polymers/lipids complexes. The sequestered anionic lipids exhibit hierarchical mobility, which is sensitively modulated by the chain rigidity and concentration of the cationic polymers. We also analyze the effects of rigidity and concentration of the anchored polymers on the restricted mobility of the polymers/monolayer complexes with the monolayer compositions of PC:PIP_2_ = 99:1 and PC:PS:PIP_2_ = 89:10:1. For both cases, we also find similar dependences of *m*_pc_, *m*_d_, and *m*_l_ on the rigidity and concentration of the anchored polymers (data not shown). The simulation results for PC:PS:PIP_2_ = 89:10:1 appear to serve as a rationale for the formation of membrane heterogeneity with realistic lipid compositions. Due to the enhanced electrostatic interactions between the cationic polymers and the monolayer, *m*_pc_, *m*_d_, and *m*_l_ slightly decrease compared with the results for PC:PS:PIP_2_ = 98:1:1. Finally, we explore the salt effect of the solution on the polymers/monolayer interactions, and we find that the screening effect of the solution with higher salt concentrations can drastically weaken the polymer anchoring and lipid sequestration. Under this condition, the anchored polymers, lipid clusters, and single sequestered lipids exhibit faster motilities. Our results are thus consistent with previous experiments showing that the extent of the PIP_2_ sequestration decreases as the salt concentration increases [8].

## 4. Conclusions

In this work, we have explored the interactions between anchored cationic polymers and a mixed fluid lipid monolayer composed of charge-neutral, univalent, and tetravalent anionic lipids using Monte Carlo simulations. In particular, we systematically examine the effects of the chain rigidity and concentration of polymers on the spatial sequestration and mobility heterogeneity of different anionic lipids. Our simulation results illustrate that the tetravalent anionic PIP_2_ lipids preferentially sequester around the anchored cationic polymers, and migrate with polymers together on the monolayer. Increasing the polymer chain intrinsic rigidity enlarges the demixing entropy loss of the sequestered anionic lipids but diminishes the conformational entropy loss of the anchored cationic polymers. Due to this energy–entropy competition, the polymers/monolayer interaction strength exhibits a non-monotonic dependence on the chain rigidity at low polymer concentrations. Polymers with intermediate rigidity exhibit the maximal anchoring ability on the monolayer, which results in the formation of larger sequestered PIP_2_ clusters and slower mobility of the polymer/PIP_2_ complexes. On the other hand, the enhanced inter-polymer competitions result in reduced electrostatic energy gains for each polymer/lipids complex at sufficiently high polymer concentrations. Under such conditions, the polymers/monolayer interaction strength monotonically decreases with increasing chain rigidity. The anchored flexible polymers can confine nearly all the tetravalent PIP_2_ lipids and further sequester the univalent PS lipids around the polymer/PIP_2_ complexes, whereas the stiffer polymers tend to partially dissociate from the monolayer and only sequester smaller PIP_2_ clusters with an increased mobility. We further illustrate that the mobility gradient of the single PIP_2_ lipids is sensitively modulated by the cooperative effects between anchored segments of the polymers in the sequestered lipid clusters. Therefore, our work demonstrates that the structural and dynamical properties of the anionic lipids can be regulated by adjusting the rigidity and concentration of the anchored polymers.

## Figures and Tables

**Table 1:** Persistence length *L*_p_ (Å) of the cationic polymers for various chain rigidity parameters *κ* at different salt concentrations *C*_i_.

*L*_p_ [Å]					
*κ*	0	10	20	50	100
*C*_i_ = 0.03 M	39.4	44.0	48.5	55.3	59.1
*C*_i_ = 0.01 M	47.4	49.5	51.2	56.8	60.7
*C*_i_ = 0.001 M	51.7	58.5	60.1	62.6	67.7

## Abbreviations

The following abbreviations are used in this manuscript:

PC: Phosphatidyl-choline
PS: Phosphatidylserine
PIP_2_: Phosphatidylinositol 4,5-bisphosphate
MC: Monte Carlo
MSD: Mean-square-displacement
RDF: Radial distribution function
BAC: Bond angle correlation function
*L*_p_: Persistence length
*l*_B_: Bjerrum length
*l_D_^−^*: Debye screening length
*κ*: Parameter to tailor the degree of polymer chain rigidity
*C*_p_: Polymer concentration
φ_s_: Fraction of the sequestered lipids in the interaction zones of all anchored polymers
*M*_s_: Number of the sequestered lipids in the zones underneath each anchored polymer
*g*_PIP2_(*r*): Radial distribution function for PIP_2_–PIP_2_ lipids
*g*_PS_(*r*): Radial distribution function for PS–PS lipids
*g*_s_(*r*): Distribution of the segments of the anchored polymers above the monolayer surface
*m*_pc_: MSD of the center of mass of the anchored polymers
*m*_pd_: MSD of lipid micro-domain
*m*_pl_: MSD of single sequestered lipids
